# A Study on the Disclosure of People with Mental Illness

**DOI:** 10.1192/j.eurpsy.2024.358

**Published:** 2024-08-27

**Authors:** M. Seo, M. H. Lee, Y. R. Kim

**Affiliations:** ^1^Gyeongsang National University, Jinju; ^2^Mokpo National University, Mokpo, Korea, Republic Of

## Abstract

**Introduction:**

People with mental illness often experience a concealable stigmatized identity that may be invisible to others. As a result, they are often faced with the dilemmas of whether to disclosure or conceal their diagnosis and their experience. However, in order to overcome the social stigma and self-stigma that hinder their recovery, they must establish a network and social support through identity disclosure.

**Objectives:**

This study investigates the effect of clinical characteristics (symptom and social function level), self-stigma and social support on the disclosure of people with mental illness.

**Methods:**

The research was conducted with 236 respondents who are currently using community mental health services. (Male: 51.9%, Female: 48.1%; Mean age = 47.97±13.24; SPR: 66.8%, other diagnosis: 33.2%).

**Results:**

Most respondents disclosed their mental illness to health service providers and family, but they are least open about their identity toward neighbors and co-workers. A regression analysis of predictors of disclosure revealed that only social functioning level and social support had significant predictive power. It was discovered that individuals with better level of social function and social support disclosure more about their mental illness.

**Conclusions:**

A program that increases social functions and support network can be recommended to improve disclosure efficacy.

**Disclosure of Interest:**

None Declared

